# Hybrid Perovskite-Based Materials Modified with Polyhedral Silsesquioxanes—Structure and Properties

**DOI:** 10.3390/ma16196531

**Published:** 2023-10-01

**Authors:** Anna Kowalewska, Kamila Majewska-Smolarek

**Affiliations:** Centre of Molecular and Macromolecular Studies, Polish Academy of Sciences, Sienkiewicza 112, 90-363 Łódź, Poland; kamila.majewska-smolarek@cbmm.lodz.pl

**Keywords:** perovskite, silsesquioxane, POSS, hybrid materials

## Abstract

Polyhedral oligomeric silsesquioxanes (POSS) and hybrid organo-halide perovskites are two important types of hybrid nanoscale frameworks with great potential in materials chemistry. Both are currently under intensive investigation for a wide range of possible applications. Recent results suggest that POSS can be attractive passivating and structure-controlling agents for perovskite materials. In this review, we present the importance of POSS in engineering the structures of inorganic cesium-halide perovskites CsPbX_3_ (X = Cl, Br, I) to create a new class of hybrid derivatives with improved properties. The combination of these two components can be an effective strategy for controlling the perovskite crystallization process. In addition, passivation of surface defects/bulk and the engineering of energy and optoelectronic properties of perovskite-based materials can be achieved following this method. In this minireview, we summarized the existing literature reports on the structural specificity and properties of hybrid POSS perovskites.

## 1. Introduction

**Scheme 1:**
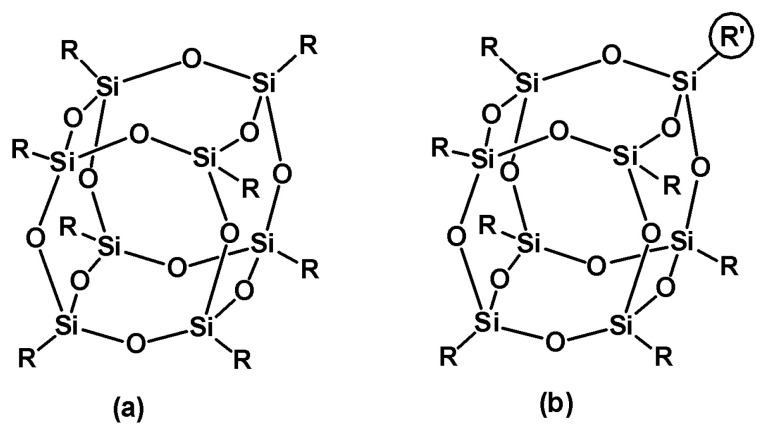
Chemical structure of (**a**) monofunctional (RSiO_3/2_)_8_ with functional the group R and (**b**) bifunctional POSS (RSiO_3/2_)_7_(R′SiO_3/2_)_1_ where R can be iBu or Ph and R′ is a specific organic substituent.

Polyhedral oligomeric silsesquioxanes (POSS) of general the chemical composition (RSiO_3/2_)_n_ are an important and extensively studied class of hybrid nanoscale materials. While the inorganic cage of the best-known POSS is usually octahedral, the chemical structure of organic functional groups R on the silicon atoms may vary (Scheme 1) [1]. The unique structure of POSS makes them a versatile component of various organic-inorganic hybrid materials. Functionalized POSS were thus applied for the synthesis of inorganic complexes and catalysts [2,3,4,5,6], including extended MOF architectures [7,8] as well as photoactive [9], self-healing [10] and self-assembling systems [11], hybrid polymer composites [12,13,14,15,16,17], and materials for biomedical applications [18,19,20,21].

Interesting results obtained in the field of materials engineering have inspired the use of polyhedral silsesquioxanes to improve the structure and properties of hybrid organo-halide perovskites. Materials based on this type of perovskite material are of great interest in the field of organic electronics, both in basic research and in a wide range of applications [22,23,24,25]. Techniques for their preparation are facile and relatively inexpensive, and thus they offer significant potential for thin-film solar cell technologies. Their characteristic structural features are sheets of organic and inorganic components alternately stacked on the molecular scale [26]. Alkylammonium cations are often used as layer separators in metal halide perovskites, acting as a barrier against magnetic and electronic coupling. Three-dimensional perovskites with the formula ABX_3_ (A: amine, B: metal, and X: halide) are typically obtained when small organic amine molecules are used as the precursors of cationic species, whereas A_2_BX_4_ two-dimensional perovskite structures are produced when larger amine molecules are employed.

Inorganic cesium lead halide CsPbX_3_ (X = Cl, Br, and I or mixed halide) perovskite nanocrystals (NCs) are dominant in this field, owing to their high photoluminescence quantum yield (PLQY, up to 90% in solution) and narrow emission peaks, as well as their easy solution processing [27]. The exceptional optoelectronic characteristics of CsPbX_3_ NCs, and their photovoltaic performance, make them valuable emissive materials in electroluminescent (EL) devices, including light emitting diodes (LED), solar cells, lasers, and photodetectors [28]. Small intrinsic defects do not act as electronic trap states [29], and their characteristic emission colors can be adjusted across the visible range by tuning the size of NCs and their chemical features [30,31,32,33]. Nevertheless, reducing the density of defect-induced traps is still required to further improve the photovoltaic performance and stability of CsPbX_3_ NCs. The ionic nature of perovskite NCs makes their ligands labile and sensitive to polar reagents as well as halide anion exchange reactions. Thus, new strategies for adjusting the crystallization kinetics and modifying the grain boundaries during rapid crystal growth in solution-processed perovskite thin films are constantly being sought to control crystallographic defects.

The use of various Lewis acids and bases, ammonium salts, and ionic liquids as additives can be a method to influence the perovskite crystallization process, the passivation of surface/bulk defects, and the engineering of their energetic and optoelectronic properties, with particular emphasis on the improvement and stabilization of their photoluminescence quantum yields [34]. Recent results suggest that POSS can be attractive passivating and structure-controlling agents for perovskite materials. In this minireview, we describe the role that polyhedral silsesquioxanes can play in terms of the impact of POSS on the structural engineering of organic-halide perovskites, the enhancement of the photovoltaic performance, as well as the properties and stability of these photoactive materials. Despite the well-known advantages of polyhedral silsesquioxanes and CsPbX_3_-based systems, the literature on hybrid POSS-perovskite compositions is, surprisingly, not very extensive. However, the published reports point to the very interesting properties of such combinations, which, if properly designed, may open up new areas of application. With this in mind, we hope that this review of the existing data will renew, inspire, and stimulate new trends in materials chemistry.

## 2. Discussion

### 2.1. Layered POSS-Metal Halide Hybrid Perovskites

**Scheme 2:**
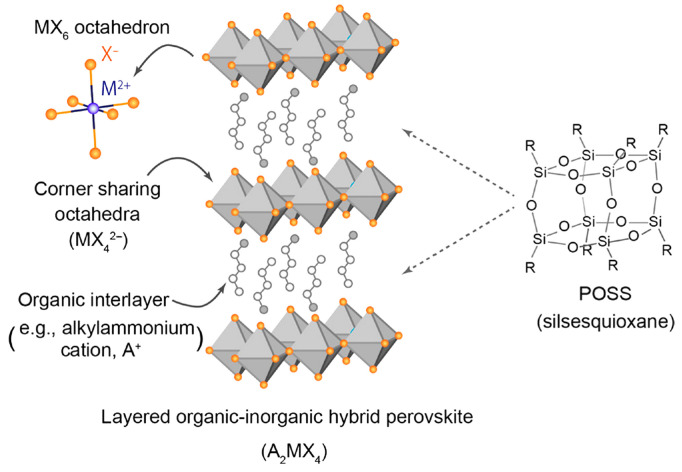
Structural features of layered organic-inorganic hybrid perovskites and POSS. Reprinted with permission from Ref. [41]. Copyright (2015) American Chemical Society.

The interfacial layers play an important role in determining the efficiency and stability of perovskite structures, and various strategies have been developed to cope with the problem of surface engineering [35]. The successful application of functionalized trialkoxysilanes for the formation of siloxane networks between perovskite layers and interface passivation was reported [36,37,38,39,40]. Cage-like POSS molecules, that exhibit a characteristic tendency to self-organize, can also be a suitable interlayer component of perovskite structures (Scheme 2). The advantage of POSS over the random silsesquioxane networks obtained in the sol-gel process with trialkoxysilanes is the uniform morphology of the inorganic layer due to the defined size of silsesquioxane nanocages.

A new class of low-dimensional layered POSS-metal halide hybrid perovskites was prepared under ambient conditions using octa(ammoniumpropyl)octasilsesquioxane chloride (A-POSS) and several metal halide salts (CuCl_2_, PdCl_2_, PbCl_2_, and MnCl_2_) [41]. The components were first dissolved in aqueous hydrochloric acid or water, and then the complexes were isolated as microcrystalline precipitates. The structural features of the hybrid complexes indicate the dependence of the stability of octahedral POSS on the synthesis conditions. The cage silsesquioxane structure mostly remained intact in the complexes, with metal halides formed in the process. Solid-state ^29^Si MAS NMR recorded for the hybrid species showed a slight upfield shift of the peak attributed to the T^3^-type silicon atoms in the cubic POSS structure. The extent of the shift depended on the type of perovskite cation (δSi = −63.9 ppm; −67.1 ppm; −65.1 ppm; −64.4 ppm; and −64.1 ppm for, respectively, A-POSS, Cu-A-POSS; Pd-A-POSS; Pb-A-POSS; and Mn-A-POSS). A slight peak broadening was attributed to the magnetic effect of the metal halide layers, especially in the case of the Cu and Mn complexes. For Pb-A-POSS, the broadening of the main peak was accompanied by the appearance of a weak resonance peak around −55 ppm. The chemical shift range indicates that the band may have arisen from the partial degradation of the silsesquioxane cage. Pd-A-POSS was an exception, as their ^29^Si NMR spectra featured a sharp T^3^ peak.

**Figure 1:**
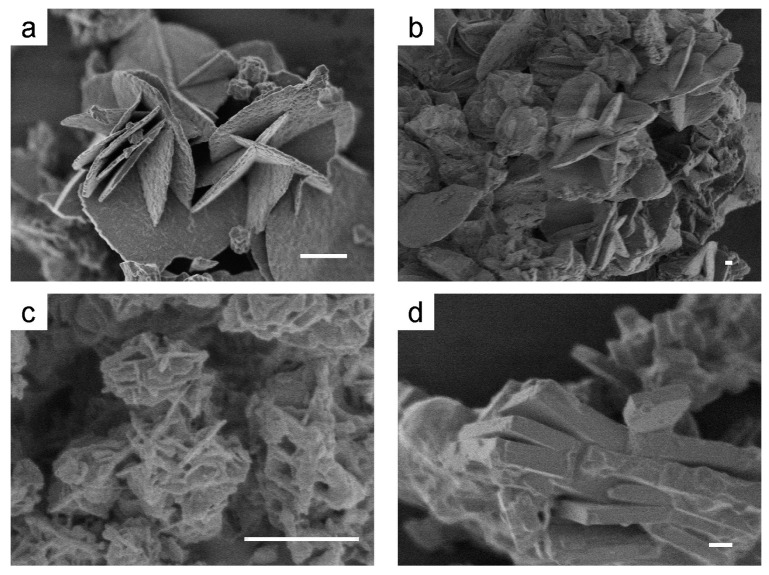
FE-SEM images of Cu-A-POSS (**a**); Pd-A-POSS (**b**); Pb-A-POSS (**c**); and Mn-A-POSS (**d**) (scale bar: 1 μm). Reprinted with permission from Ref. [41]. Copyright (2015) American Chemical Society.

All the prepared hybrid complexes precipitated as thin plates, but their specific morphology was characteristic of the type of metal halide used (Figure 1). Mn-A-POSS crystallized as thick rectangular plates, while the Cu-A-POSS and Pd-A-POSS crystals were disc-shaped with sharp edges. The Pb-A-POSS crystals were also disc-shaped, but much less defined. As the native A-POSS precursor did not crystallize in such a form, the observed morphology was assigned as originating from the layered perovskite structures.

In line with this, the powder XRD measurements evidenced the high crystallinity and characteristic reflections of the perovskites in the self-assembled layered products. The layer inter-distance (Cu-A-POSS: 1.76 nm; Pd-A-POSS: 1.70 nm; Pb-A-POSS: 1.61 nm; and Mn-A-POSS: 1.74 nm) corresponded to the size of A-POSS (~1.4 nm [42]). The silsesquioxane molecules were located between the layers of the perovskite NCs and were the cause of the formation of micropores, as evidenced by the N_2_ sorption isotherms for all the hybrid materials obtained, except Pb-A-POSS. In this particular case, the inter-layer distance was too low, possibly due to the partial degradation of the POSS structures, as evidenced by the ^29^Si NMR measurements. Cu-A-POSS and Pd-A-POSS exhibited type-I N_2_ sorption isotherms (average micropore diameters <2 nm and their BET specific surface areas were 205 and 187 m^2^/g, respectively). The physical performance studies demonstrated that the A-POSS interlayers improved the low-dimensional properties of the hybrid materials. Cu-A-POSS and Mn-A-POSS showed different magnetic ordering in their perovskite layers (Cu-A-POSS: ferromagnetic ordering and temperature dependence of the molar susceptibility (χmol) with a maximum around 5 K; Mn-A-POSS: antiferromagnetic interactions between Mn^2+^ ions).

Interestingly, the use of octa(propylammonium)octasilsesquioxane bromide (Br-POSS) or iodide (I-POSS) for the co-crystallization with PbCl_2_ did not involve the degradation of the silsesquioxane cube [43]. The plate-like products precipitated quite rapidly, and their PXRD diffractograms indicated layered structures with interlayer spacings (1.61 nm and 1.62 nm, respectively) similar to those calculated for Pb-A-POSS. The ionic silsesquioxane interlayers stabilized the excited electron-hole pairs in the semiconducting Pb-X-POSS lead halide perovskites (X = Cl^−^NH_3_^+^; Br^−^H^+^; I^−^H^+^) with well isolated layers, even if they lacked porosity. This effect is important for potential applications of hybrid POSS-perovskite materials as light-emitting diodes and light absorbers in solar cells. The absorption and the exciton emission peak positions shifted towards longer wavelengths by changing the ionic group from Cl^−^NH_3_^+^ to Br^−^H^+^ to I^−^H^+^. For Pb-Br-POSS and Pb-I-POSS, strong absorption bands were observed at ~390 and 510 nm, while sharp PL peaks appeared at 424 and 526 nm, respectively, when excited with *λ*_ex_ = 355 nm. These results showed that by selecting the composition of hybrid complexes, it is possible to adjust both the structure and the properties of NCs. This may have a significant impact on the design process of, e.g., solar cell technology.

**Scheme 3:**
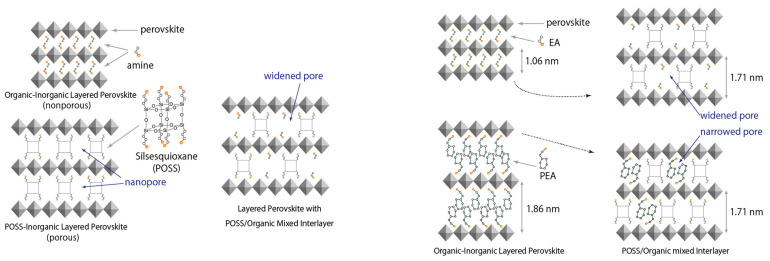
Schematic morphology of hybrid perovskites with mixed interlayers—illustration trends related to amine molecular size (expansion or shrinking of micropores). Reprinted with permission from Ref. [44]. Copyright (2018) American Chemical Society.

It was also postulated that the dielectric properties of the interlayers could be tuned by filling the micropores with different chemicals. The addition of organic amines (replacement of up to 20% of the silsesquioxane interlayers) caused changes in the porosity and the physical properties of Cu-A-POSS without changing the interlayer distance characteristic of A-POSS (Scheme 3) [44]. Alkylamines of small molecular size (e.g., ethylamine; EA) caused up to a 44% increase in the micropore volume, while the more bulky amines (e.g., phenethylamine; PEA), surprisingly, induced pore shrinking by as much as 43%. It was also found that the range of the micropore volume can be adjusted by changing the ratio of the amine fraction.

The observed phenomena were explained by detailed structural analysis. The PXRD patterns showed differences between the interlayer thicknesses of the perovskites obtained from EA (1.06 nm) or PEA (1.86 nm) alone and those containing a mixture of one of the amines and A-POSS (1.71 nm regardless of the type of organic amine). Increasing the amount of EA resulted in a product of bimodal structure with the layer distances of 1.06 nm (EA) and 1.71 nm (A-POSS or a mixture of A-POSS and EA), as indicated by the characteristic diffraction patterns. The results of the N_2_ sorption studies corroborated the PXRD data. All the hybrid materials containing A-POSS (also those admixed with EA) contained micropores smaller than 2 nm and sorption/desorption isotherms of type I were obtained. The amount of adsorbed N_2_ was greater for Cu-A-POSS/EA than Cu-A-POSS, and the micropore volume and BET surface increased linearly with the replacement ratio (x) of POSS with EA(44% increment if x = 0.202). When x was >20%, the micropore volume and BET surface area decreased and an increase in the fraction of nonporous layered perovskites was observed with PXRD (EA-derived layered perovskites are nonporous). On the contrary, the shrinkage of the interlayer pores was observed in the case of the layered perovskites separated by mixtures of the cubic silsesquioxanes and PEA (comparing to Cu-PEA). It implies the influence of π–π stacking of the aromatic amine molecules on the porosity of the layered perovskites. The reduction in the interlayer distance suggests that the ordering of PEA was altered in the presence of A-POSS. The effect allows for the precise structural engineering of the interlayer distance and, correspondingly, the properties of such complex systems.

The diffraction patterns of samples containing A-POSS and PEA (<8.8%) resembled those obtained with A-POSS only (molecules of PEA were confined in the small space created by A-POSS). In this case, the type of N_2_ adsorption isotherm was not altered, but the amount of N_2_ adsorbed (as well as the micropore volume and the BET surface area) was smaller and it even slightly decreased as the PEA fraction increased. This general trend was supported by the results obtained with the other amines. The micropore volume increased when A-POSS was partly replaced with the small organic amines (EA, butylamine, or cyclohexylamine) and decreased when the bulky aromatic amines (PEA or 4-phenylbutylamine) were used. Despite the changes in the structure of the silsesquioxane interlayers, the magnetic moment measurements proved that the hybrid layered perovskites retain the ferromagnetic ordering at T < 20 K. Thus, the addition of the organic amines did not cause defects in the perovskite layers, but by adjusting their type and amount, desired and interesting structural changes can be achieved.

### 2.2. Encapsulation with POSS to Increase Brightness and Stability of Perovskites

Perovskite nanocrystals of the CsPbX_3_ structure are promising and valuable emissive materials in electroluminescent devices. However, the quality of an as-prepared film of NCs may be relatively poor due to the presence of the long-chain surface ligands used during the coating process, which hampers the efficiency of the charge injection. The processing problems may lead to uneven, patchy film coverage over the device sublayers. Perovskite nanocrystals can be protected from moisture by encapsulation in a hydrophobic polymer matrix, which provides a physical barrier [45,46,47,48]. However, the formation of stable perovskite-polymer composites is not trivial due to the phenomena of phase separation and nanocrystal agglomeration.

**Figure 2:**
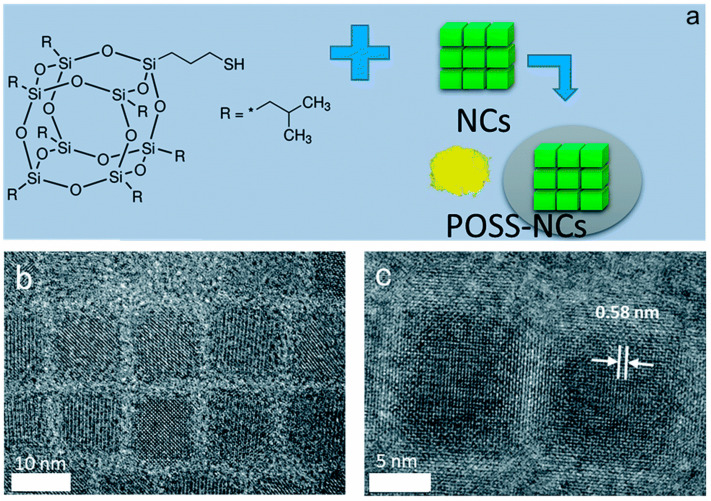
Coating of perovskite NCs with thiol-functionalized POSS (**a**), and HRTEM images of CsPbBr_3_ perovskite NCs before (**b**) and after (**c**) coating with POSS. Reproduced from Ref. [49] with permission from the Royal Society of Chemistry.

Functionalized POSS can be a solution in the engineering of perovskite materials to enhance the low stability of the ionic nanocrystal lattice. Hydrophobic polyhedral silsesquioxanes of the (RSiO_3/2_)_7_(R′SiO_3/2_)_1_ (R = i-Bu and R′ a functional group) structure can play a beneficial role as materials that can encapsulate the perovskite NCs or form an intermediate hydrophobic passivation layer for their thin films. Monofunctional POSS of this type can improve the surface coverage and the morphological features of the perovskite films and possibly improve their miscibility with polymer matrices. For example, molecules of (iBuSiO_3/2_)_7_[HS(CH_2_)_3_SiO_3/2_] were used as a surface protecting additive to the CsPbX_3_ (X = Br or I) nanocrystals (Figure 2) [49,50]. However, low amounts of POSS were required since the silsesquioxane cages may act as insulators.

**Figure 3:**
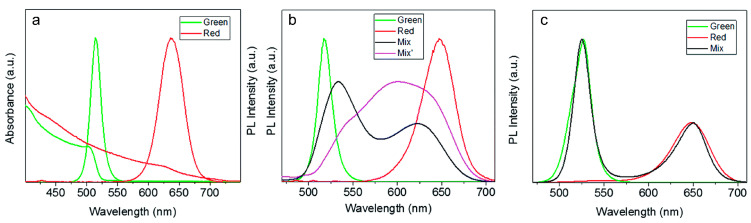
(**a**) UV-vis absorption and PL spectra of CsPbX_3_ PQD coated with POSS (─ X = Br; ─ X = Br/I). (**b**) Solid state PL spectra of non-capped CsPbX_3_ NC powders, and of their 1 : 1 molar% mixture taken immediately after mixing (Mix) and after 5 min (Mix′). (**c**) Solid state PL of POSS-capped CsPbX_3_ and of their 1 : 1 molar% mixture (Mix). Reproduced from Ref. [49] with permission from the Royal Society of Chemistry.

The treatment provided the moisture resistant hybrid perovskite nanopowders-quantum dots (PQD) that can be used as solid state luminophores in all-perovskite white light-emitting devices. The hybrid silsesquioxane molecules acted as a hole-blocking layer between the thin coating made of the perovskite NCs and the 1,3,5-tris(N-phenylbenzimidazol-2-yl) benzene (TPBi) film that operated as the electron-transporting layer. The POSS-PQD exhibited (HRTEM and PXRD; Figure 2) the lattice plane distance of 0.58 nm, characteristic for cubic phase CsPbBr_3_ perovskite, and high output performance. Moreover, the silsesquioxane coating prevented anion exchange between the perovskite nanocrystals in the solid state, thereby increasing the stability of the mixtures of the perovskite NC powders with the different halide compositions. The distinct emission spectra of the different POSS-passivated CsPbX_3_ were preserved, while the uncoated NCs underwent ion exchange, resulting in a broadening of their characteristic PL signals in the solid state (Figure 3). It should be noted that their PLQY in toluene solutions slightly decreased to 62% (X = Br) and 45% (X = Br/I) upon passivation with POSS, but the absolute PLQY in the solid state did not change and remained very high (respectively, 61% and 45%).

**Figure 4:**
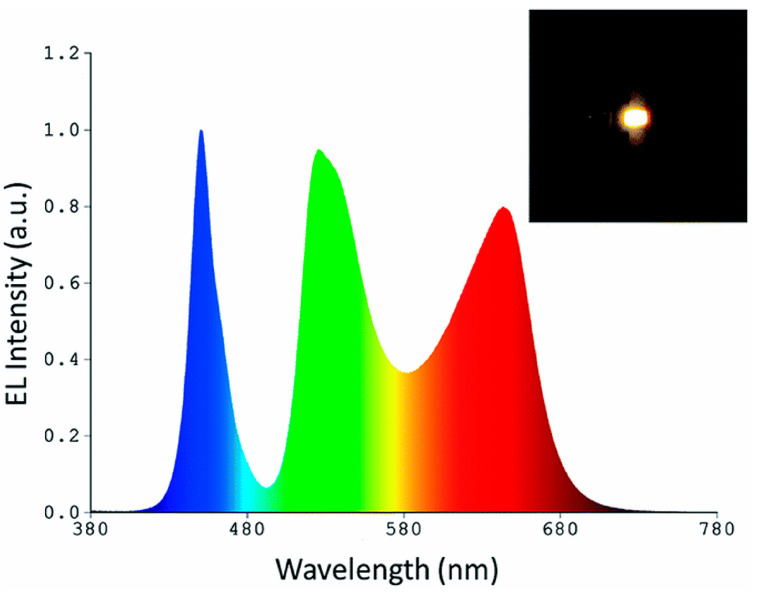
The EL spectrum of a white LED fabricated from a single-layer of mixed deposited on InGaN chip (inset: WLED operating at 20 mA). Reproduced from Ref. [49] with permission from the Royal Society of Chemistry.

This strategy yielded single layer, all-perovskite devices that emitted white light by mixing the nanopowders of the green-emitting POSS-CsPbBr_3_ and the red-emitting POSS-CsPb(Br/I)_3_. The POSS-passivated perovskites were thus used as solid state luminophores for the fabrication of all-perovskite (a single down-conversion layer) white LEDs with a CIE chromaticity coordinate of (0.349, 0.383), CRI = 81, and luminous efficiency of 14.1 lm W^−1^. The characteristic electroluminescence spectrum (Figure 4) is a combination of the three emission peaks (the blue one originated from the blue-emitting InGaN LED chip).

**Figure 5:**
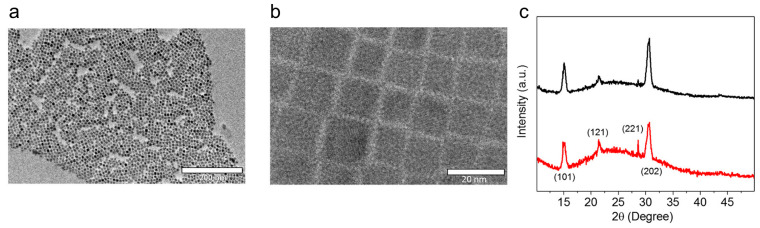
TEM images of CsPbBr_3_ NCs before (**a**) and after (**b**) modification with POSS NCs; (**c**) XRD traces from spin-coated films of perovskite NCs suspension: native (─) and after treatment with POSS (─). Reprinted with permission from Ref. [50]. Copyright (2016) American Chemical Society.

A similar approach was aimed at the enhancement of the stability and performance of the CsPbBr_3_-based electroluminescent green light-emitting devices [50]. The molecules of (iBuSiO_3/2_)_7_[HS(CH_2_)_3_SiO_3/2_] attached onto the surface of the NCs and, at larger concentrations of CsPbX_3_, separated the perovskite crystals in the suspension, prohibiting the formation of larger clusters by breaking the inter-NC attachments. The presence of POSS did not influence the absorption and PL spectra of the NCs, nor altered the crystal structure and shape of the monodisperse cube-shaped CsPbBr_3_ nanoparticles, as evidenced by the respective XRD patterns and the TEM images (Figure 5). Without POSS, the perovskite NCs formed large domains separated by domains of poorly conductive ligands, while the addition of POSS improved the substrate coverage.

The improvement in the morphology and coverage of the CsPbBr_3_ NCs in the presence of POSS did not always lead to luminance enhancement. If the POSS molecules were present in the active layer, they acted as insulators. As a result, the external quantum efficiency (EQE) value was not high and the average PL lifetimes were reduced (from 434 to 134 ns, and from 115 to 63 ns for the suspension and supernatant solutions, respectively). Nevertheless, with the optimized POSS concentrations, higher loading of the separated NCs resulted in an increase in the overall LED brightness.

It was more beneficial to use POSS as a separate layer on top of the active perovskite NC layer. In this case, the average PL decay time only decreased from 434 to 342 ns, and the average recombination rate increased by 21%. The effect of the upper POSS layer was attributed to the more efficient electron and hole recombination in the NCs zone and the blocking of hole transport between the perovskite NCs and the TPBi layers. In this case, the peak LED luminance was almost eight times higher (2983 cd/m^2^ at 11.5 V; LE = 1.20 cd/A; EQE = 0.35%) than that obtained without the hole-blocking layer. In addition, POSS enhanced the stability of the LED devices and their operation lifetime was five times longer.

**Scheme 4:**
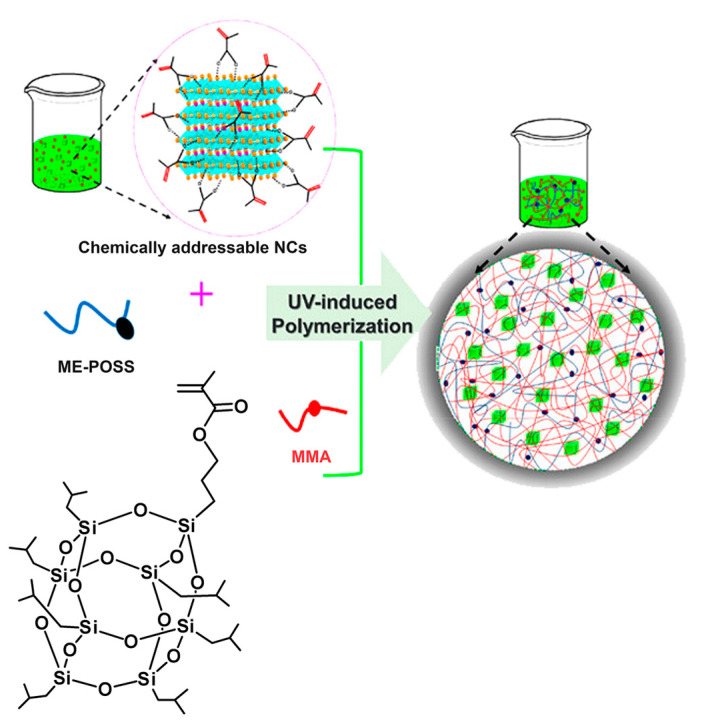
Synthesis of hybrid composites of CsPbBr_3_ NCs, POSS-ME, and poly(methyl methacrylate) (PMPOPNC). Reprinted with permission from Ref. [51]. Copyright (2018) American Chemical Society.

**Figure 6:**
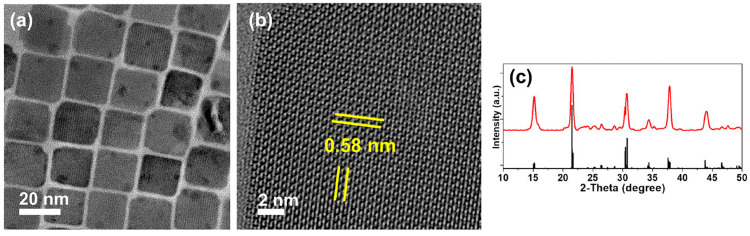
(**a**) TEM and (**b**) HR-TEM images of the ME-NCs (*d*-spacing of 5.8 Å representing (001) the crystal lattice planes of orthorhombic crystal phase); (**c**) comparison of X-ray diffraction patterns of ME-NCs (─) and orthorhombic CsPbBr_3_ (─). Reprinted with permission from Ref. [51]. Copyright (2018) American Chemical Society.

The surface of the CsPbBr_3_ NCs can be passivated through the coordination of atoms in the top layer with the carboxylate groups of (iBuSiO_3/2_)_7_[(H_3_C)H_2_C=CC(O)O(CH_2_)_3_SiO_3/2_] bearing methacrylic acid ligands (POSS-ME) (Scheme 4) [51]. The TEM micrographs of the resulting methacrylate-functionalized nanoparticles (ME-NCs) displayed well-defined particles that are larger (14-17 nm) than the typical CsPbBr_3_ NCs obtained with small organic ligands (8–11 nm, [30]), whereas the HRTEM and XRD measurements suggest the formation of an orthorhombic crystal phase in the CsPbBr_3_ NCs (Figure 6).

MA-NCs can further participate in radical polymerization reactions. The copolymerization with methyl methacrylate and/or (iBuSiO_3/2_)_7_[(H_3_C)H_2_C=CC(O)O(CH_2_)_3_SiO_3/2_] yielded composite materials (PMPNC and PMPOPNC, respectively). Their diffraction patterns corresponded to that of the orthorhombic perovskite NCs crystals. The thin films cast from the dispersions of PMPOPNC in the organic solvents contained well-dispersed crystalline nanoparticles with a size of ∼12 nm, embedded within the amorphous PMMA-*co*-P(ME-POSS) (PMPO) matrix. No phase separation was observed. The nanoparticles of PMPOPNC were larger (average size 68 nm) than the free ME-NCs and their size distribution was wider. The PL spectra of PMPNC and PMPOPNC corroborated those of the free ME-NCs and the ME-NC/PMMA composites (λ_e_ at 516 nm). Their PLQY (respectively, 68% and 72%) was similar to that of the free ME-NCs (PLQY above 80%) and larger than the PLQY of the MENCs/PMMA blend (ca. 54%). The emission wavelength and PLQY of the PMPOPNC can be tuned by adjusting the ratio of halides in the perovskite structure CsPbX_3_ by using different lead halide precursors.

The PMPOPNC film was hydrophobic (static water contact angle of 120°, comparing to 105° of PMPNC and 104° of ME-NCs/PMMA blend). The effect was assigned to the micro-nanorough features revealed in the SEM micrographs, caused by the migration of the POSS-containing fraction to the air-solid interface (confirmed by XPS gradient concentration data). In addition, the PMPOPNC films were not prone to anions exchange. Remarkably, PMPOPNC retained its luminescence over the three cycles of heating to 120 °C (followed cooling to room temperature) much better (PLQY > 80% of the initial PL intensity) than the ME-NCs, which degraded significantly (PLQY < 40%) upon the thermal treatment. The photostability of PMPOPNC under blue light was also improved (∼81% of the initial PL intensity after 156 h) compared to the native NCs. The enhanced hydrolytic resistance, thermal stability, and good solubility in organic solvents of PMPOPNC makes it potentially suitable for use as a solution-processable luminescent ink and as a green fluorophore for the fabrication of white LEDs.

### 2.3. POSS As a Moisture Barrier in Perovskite Films

As was mentioned in the previous section, apart from the employment of the POSS coated perovskite NCs as solid state luminophores, the large size of the POSS macromolecules and the presence of hydrophobic organic groups grafted to Si atoms can be an advantage with regard to their barrier action against moisture. The significant potential in this area is offered by monofunctional polyhedral silsesquioxanes of type (RSiO_3/2_)_7_(R′SiO_3/2_), where R = iBu and R′ is a reactive organic residue that enables the adsorption of POSS on the surface of perovskite crystals or thin films. The passivated perovskites can be applied as water resistant light-emitting materials.

As was mentioned in Section 2.2., the surface-capping of the CsPbX_3_ (X = Br or I) perovskite NCs by (iBuSiO_3/2_)_7_[HS(CH_2_)_3_SiO_3/2_] (POSS-SH) containing a thiol group resulted in an increase in the water resistance and anion exchange prevention, both in water and in the solid state [49]. The POSS-CsPbBr_3_ NC powder was stable after dispersion in water and the suspension that was formed emitted intensive green light even after 10 weeks of storage. Moreover, the PL characteristics of the aqueous suspension did not change on the treatment with HI.

It was also shown that [3-(2-aminoethyl)amino]propyl-heptaisobutyl substituted POSS (iBuSiO_3/2_)_7_[H_2_N(CH_2_)_3_SiO_3/2_] (POSS-NH_2_) can be applied as a capping ligand for (CH_3_NH_3_)PbBr_3_ (MAPbBr_3_) (MA—methylammonium) [52]. POSS-NH_2_ passivated the surface of the MAPbBr_3_ NCs, controlling the crystal size and increasing the perovskite material stability in the LED devices. The importance of the presence of sterically demanding POSS ligands was demonstrated by the comparison of the structure and properties of hybrid NCs with their analogs modified with (3-aminopropyl)triethoxysilane (APTES) as the caping ligands. As a result of the presence of the silsesqioxane layer and the increased steric hindrance, both the APTES- and the POSS-NH_2_-grafted NCs showed higher stability in protic solvents compared to the NCs containing straight-chain organic ligands. The hybrid NCs of different sizes (ca. 2.5–100 nm, depending on the amount of the ligand present) were uniform and showed a high photoluminescence quantum yield (~15–55%). Both the absorption and the emission bands of the modified hybrid NCs blue-shifted when the concentration of the capping agents increased.

**Figure 7:**
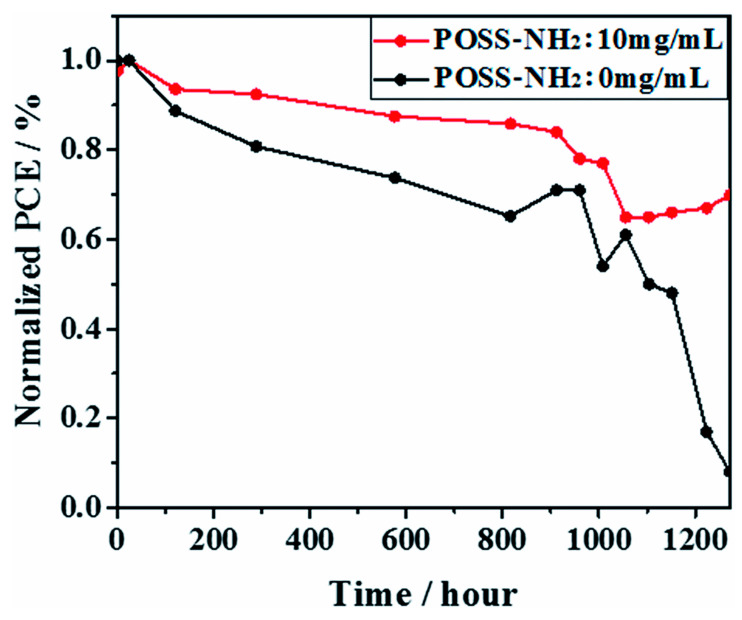
Stability comparison of perovskite solar cells with/without POSS-NH_2_ modification. Reproduced from Ref. [54] with permission from the Royal Society of Chemistry.

POSS-NH_2_ made the surface of a thin layer of the perovskite NCs waterproof, influenced the structure of the film, and helped to tune its optoelectronic behaviour [53]. The photovoltaic performance with a power conversion efficiency (PCE) over 20% was observed. It was also shown that POSS-NH_2_ can effectively passivate the surface of thin coatings of NCs composed of the mixed halide perovskites, MA*_x_*FA_1−*x*_PbI_3−*y*_Br*_y_* (FA—formamidinium), as well as influence the crystal grain boundary and the number of ionic defects [54]. The temperature-dependent admittance measurements proved that the presence of POSS-NH_2_ reduced the charge trap density and, as a result, the trap-state energy level of 0.045 eV was achieved. Those features were reflected in an enhancement of the open-circuit voltage (V_OC_) and power conversion efficiency from 18.1% to 20.5%. The photovoltaic solar cells protected by the POSS passivation layer were also more stable and only 20% degradation of the PCE in an inert atmosphere of N_2_ was noted after 1000 h (Figure 7).

**Figure 8:**
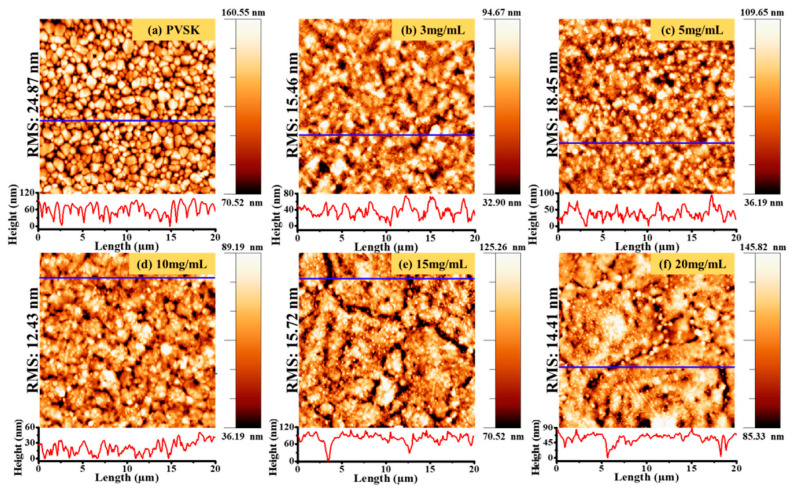
AFM images and height profiles of perovskite films passivated with POSS-NH_2_ ((**a**–**f**) increasing concentration of POSS) [55].

As was already mentioned, the presence of POSS is beneficial, but its quantity must be optimized because the inorganic cube acts also as an insulator. It was shown that the surface composition of the FA_0.85_MA_0.15_Pb(I_0.85_Br_0.15_)_3_ films and their morphology changed in a regular manner in the presence of POSS-NH_2_ as a result of its interaction with the perovskite film [55]. When the amount of silsesquioxane exceeded the optimum concentration (10 mg/mL), the quality of the perovskite film surface morphology was compromised by the formation of cracks (Figure 8). The AFM height profiles displayed by the variations in the root mean square (RMS) values show that the size of NCs changed on modification with POSS-NH_2_ to a degree that depended on the amount of POSS used for the modification. The lowest RMS (12.43 nm) was obtained for the optimal concentration of the passivating agent. The formation of cracks at higher concentrations of POSS-NH_2_ resulted in an increase in RMS. Correspondingly, a decrease in the device’s performance with regard to the open-circuit (V_OC_), fill factor (FF), and power conversion efficiency was noted. Only the short-circuit current (J_SC_) values did not decrease for the samples obtained with higher concentrations of the silsesquoxane.

**Figure 9:**
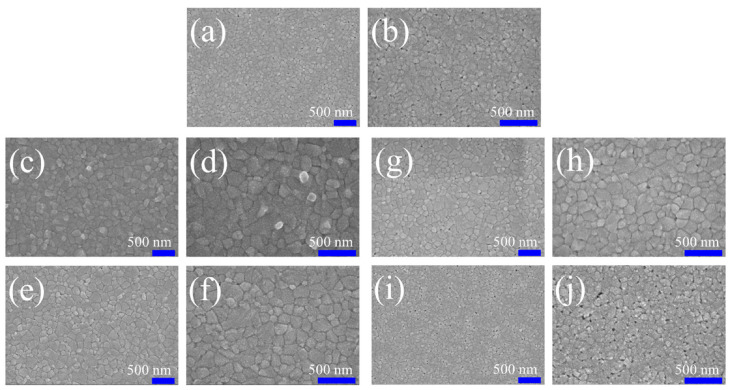
SEM images of the MAPbI_3_ thin films deposited over the FTO/NiO_X_/POSS with various POSS contents: (**a**,**b**) control; (**c**,**d**) POSS-0.005; (**e**,**f**) POSS-0.01; (**g**,**h**) POSS-0.015; and (**i**,**j**) POSS-0.05 [56].

Acryloxypropyl isobutyl POSS (iBuSiO_3/2_)_7_[H_2_C=CHC(O)O(CH_2_)_3_SiO_3/2_] was spin-coated as an ultra-thin passivation layer over the hole transporting layer of nickel-oxide (NO_x_) in a fluorine-doped tin oxide (FTO)/NO_X_/POSS/MAPbI_3_/PC_61_BM/Bathocuproine (BCP)/Ag perovskite solar cell of inverted structure [56]. The presence of the silsesquioxane layer resulted in a more hydrophobic and smoother support surface for further perovskite deposition. As a result, an increase in the grain size of perovskite was observed (Figure 9). The POSS passivation layer effectively reduced the electron/hole recombination at the grain boundaries, which brought in almost 14% increase in the short-circuit current (J_SC_ from 18.0 to 20.5 mA·cm^−2^ for the optimised POSS concentration of 0.01 mg/mL). Moreover, the V_OC_ of the cell increased slightly over 1 V. Larger amounts of POSS may result in an increase in the pinhole, which adversely affects the charge transport resistance despite an increase in the charge recombination rate.

**Scheme 5:**
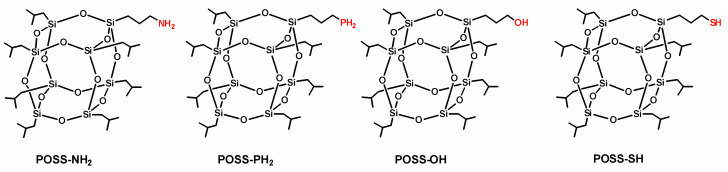
Molecular structures of POSS-NH_2_, POSS-PH_2_, POSS-OH, and POSS-SH.

Despite the general trend towards the increased stability of the perovskite NCs as a result of the treatment with functionalized POSS, the choice of reactive functional groups is crucial to the effectiveness of their surface passivation. The influence of four different functional groups R grafted to the inorganic core of (iBuSiO_3/2_)_7_[R(CH_2_)_3_SiO_3/2_], including amine (-NH_2_), phosphine (-PH_2_), hydroxyl (-OH), and thiol (-SH) residues (Scheme 5), was evaluated by density-functional theory (DFT) calculations [57]. Mechanistic theoretical studies have demonstrated the role functional groups can play in the adsorption process in relation to their specific chemical properties.

**Scheme 6:**
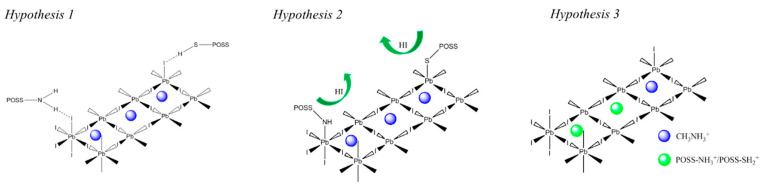
Three hypothetical molecular models of surface passivation with (iBuSiO_3/2_)_7_[R(CH_2_)_3_SiO_3/2_]. Reprinted with permission from Ref. [57]. Copyright (2019) American Chemical Society.

POSS-NH_2_ and POSS-SH were also experimentally tested as passivation materials for the MAPbI_3_ and (FA)_0.85_(MA)_0.15_Pb(I_3_)_0.85_(Br_3_)_0.15_ perovskite thin films in terms of both their power conversion efficiency and their long-term stability. The conclusions drawn from the measurements of wetting angle changes and X-ray diffraction data, as well as the stability of the solar cells in the ambient atmosphere, supported the results of the DFT calculations. Three different passivation mechanisms were considered to explain the observed phenomena (Scheme 6). The first hypothesis was based on neutral interactions between the functionalized POSS molecules (e.g., POSS-NH_2_) and the iodide ions on the surface of the model perovskites. It was noted that the coordination involved electrostatic interactions between the central Cs^+^ ions in the perovskite structure and the partially negatively charged atoms of nitrogen and sulfur. Moreover, the secondary interactions of the agostic C-H⋯surface type also contributed to the overall energy. The second hypothesis involved the contacts between the perovskite film surface and the anionic POSS species, resulting from the chemical reactions on the MAPbI_3_ surface with the formation of direct Pb-N (or P, O, S) bonds by the release of hydrogen iodide (HI). It was considered the least likely. The third (acid) hypothesis postulated the formation of a protonated, cationic POSS (POSS-NH_3_^+^/POSS-SH_2_^+^) that could replace the MA^+^ cations in the top layer of the MAPbI_3_ film, resulting in a passivated hydrophobic surface.

It was concluded that, in general, at this level of theory calculations, adsorption is significant and corresponds to the formation of a weak covalent bond, although secondary interactions may also play a role. Although all the POSS systems showed a consistent preference for the third hypothesis, the differences in the adsorption energies were similar for the 1st and 3rd approach. Both models of adsorption can possibly take place in parallel. The POSS with the amine function adsorbed stronger (with 86 (56) kJ mol^−1^) than the POSS-SH to the surface of the perovskite film.

**Figure 10:**
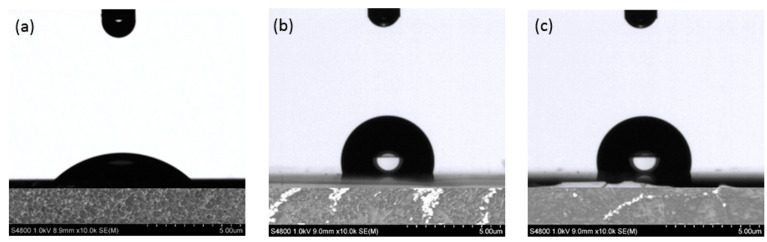
Images of drops of water and SEM top view of MAPbI_3_; films (**a**) neat; (**b**) coated with POSS-NH_2_; (**c**) coated with POSS-SH. Reprinted with permission from Ref. [57]. Copyright (2019) American Chemical Society.

The contact angle investigations revealed that the MAPbI_3_ surface that was hydrophilic (contact angle 47°) and polycrystalline (crystallite size 200−500 nm) before the passivation became more hydrophobic after both the coating with POSS-NH_2_ (contact angle 107°) and with POSS-SH (contact angle 109°) (Figure 10). The similar value of the wetting angles can be related to the presence of the hydrophobic isobutyl groups. The SEM images showed that the surface was coated with POSS molecules. Little aggregation was observed only after the entire perovskite surface was covered, indicating that the interactions between the functional groups and the perovskite surface are stronger than the POSS self-aggregation effect. The morphological observations were confirmed by the photoluminescence measurements after the passivation with POSS-NH_2_ and POSS-SH (PL peaks intensities increased and were blue-shifted). The wetting angle of the perovskite film after the POSS-NH_2_ deposition and the subsequent washing with chlorobenzene decreased to 76° (67° in the case of the POSS-SH passivated film), but was still significantly higher than that of the pristine material. The perovskite films passivated by POSS-NH_2_ showed strong repulsion of the water droplet within 40 s (6 s for POSS-SH passivated sample). This shows that POSS-NH_2_ adsorbs more strongly on the MAPbI_3_ surface than POSS-SH.

**Figure 11:**
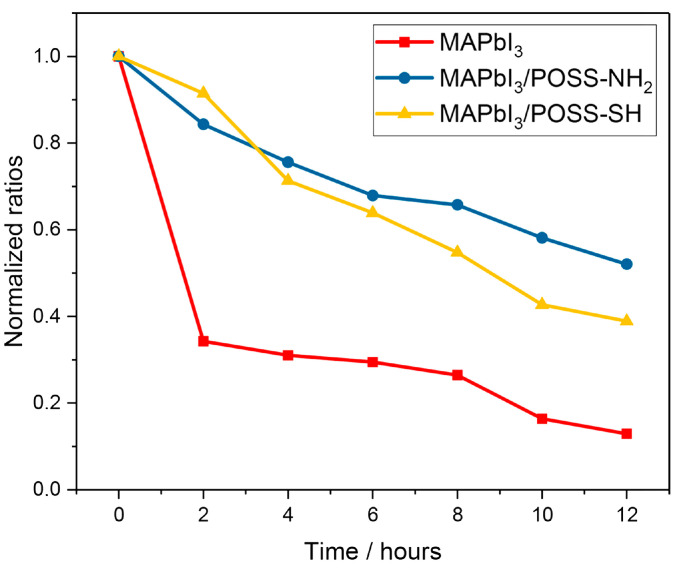
Change in normalized intensity ratios between the peaks of (110) MAPbI_3_ and (001) PbI_2_ over time at a relative humidity of (90 ± 5)%. Reprinted with permission from Ref. [57]. Copyright (2019) American Chemical Society.

X-ray diffraction was employed to follow the changes in the structure of the pristine MAPbI_3_ and the passivated films (Figure 11). The relative intensity of the peak at 2θ = 13.03° assigned to (001) crystal plane in PbI_2_ compared to the 2θ = 14.49° that corresponds to (110) crystal plane in MAPbI_3_ indicated the degree of moisture-induced degradation of the latter (resulting in the formation of PBI_2_). The increase in the intensity of the (001) peak was significantly retarded in the passivated samples. POSS-NH_2_ protected the perovskite film more efficiently than POSS-SH.

Passivation with POSS had a limited effect on the photovoltaic performance, as shown by the J-V characteristics of the MAPbI_3_-based devices. The efficiency of 16.25% was recorded for the MAPbI_3_-only specimen, while the passivated samples had efficiencies of around 16.01% and 15.89% for POSS-NH_2_ and POSS-SH, respectively. After passivation with POSS, a slight decrease in the current density (J_SC_) and open circuit voltage was observed. This effect was explained by the partial blocking of charge carriers at the interface by the bulky POSS molecules. Correspondingly, an increase in the transport resistance was found by the electrochemical impedance spectroscopy (EIS) measurements in the POSS-passivated devices. The increase in the recombination resistance and the reduction in the recombination loss at the perovskite-HTM interface was linked to the adsorption of POSS-NH_2_ on the surface of the perovskite film.

### 2.4. Application of Water-Resistant Hybrid POSS-Perovskite Systems

The stability of the MAPbI_3_-based solar cells, based on the passivated films under ambient environment, was shown by the stable V_OC_ and J_SC_ values during 90 days. After that time, the solar cells made of the perovskites passivated by POSS-NH_2_ and POSS-SH retained, respectively, 100% and 94% of their initial J_SC_ (fill factors around 89% and 88%, respectively, of their original performance and 72% of the initial FF for the non-passivated devices). The better protection offered by POSS-NH_2_ corroborated the stronger interactions of this compound with the perovskite film. The protective effect provided by POSS was less pronounced in the case of the solar cells based on (FA)_0.85_(MA)_0.15_Pb(I_3_)_0.85_(Br_3_)_0.15_ with formamidinium ions embedded in the perovskite structure. Passivation with POSS played a minor role in such stable systems, which already had good moisture resistance.

[3-(2-Aminoethyl)amino]propyl-heptaisobutyl-POSS was used for the synthesis of an amphiphilic copolymer (*ap*-POSS-PMMA-*b*-PDMAEMA, methylmethacrylate, MMA, and 2-(dimethylamino)ethylmethacrylate, DMAEMA) further applied to prepare the stable core-shell colloidal perovskite nanocrystal-polymer micelle composites (*ap*-POSS-PMMA-*b*-PDMAEMA@CsPbBr_3_) [58]. The presence of the hydrophobic POSS-PMMA segment of *ap*-POSS-PMMA-*b*-PDMAEMA was crucial in the process of self-assembling into the “reverse” micelles in DMF/toluene. The reverse micelles acted as confined nanoreactor templates during the perovskite crystallization, passivating the perovskite surface with a multidentate capping shell.

The NCs growth inside the inverted micelles proved to be an effective approach to engineering the morphology of the perovskite composites. As a result, multi-nanocubes of perovskite nanocrystal-polymer composites (6–8 nm size and PLQY > 60%) were obtained. It was demonstrated that the observed excited carrier dynamics was related to the decreased number of internal band-gap trap states. The hybrid composites were much more water-resistant than the bare NCs or the previously reported systems. After 5 h in water, the relative photoluminescence quantum yield of *ap*-POSS-PMMA-*b*-PDMAEMA@CsPbBr_3_ was about 75% of the initial PLQY, while the CsPbBr_3_ NCs were fully degraded within 0.5 h under the same conditions.

**Scheme 7:**
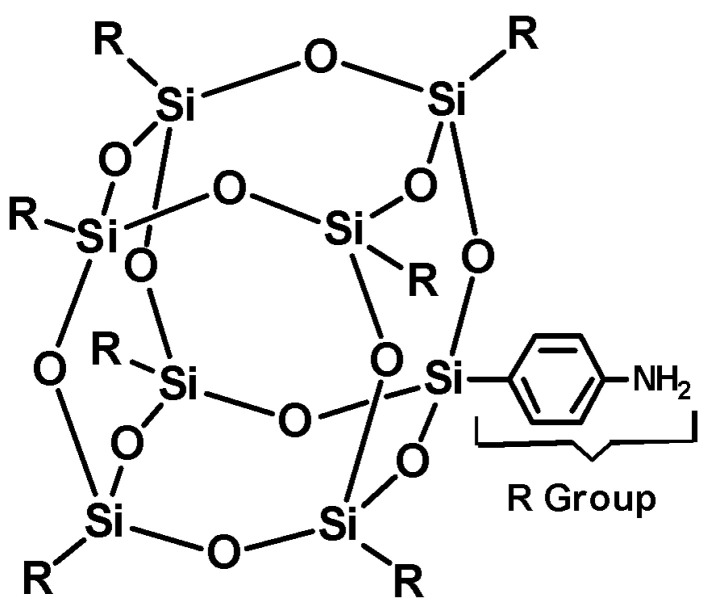
Structure of octaaminophenyl-POSS used for the preparation of aptamer-grafted hybrid perovskite quantum dots POSS-PQDs-Apt.

The photo-optical properties of the CsPbX_3_ NCs (narrow PL emission width, tunable bandgap and high quantum yield) make them good candidates for fluorescence resonance energy transfer (FRET) aptasensor probes, on the condition of adequate hydrolytic stability. Passivation of the perovskite NCs with functionalized POSS provides perovskite quantum dots with a hydrophobic shell, while not shielding the PL emission. For example, octaaminophenyl-POSS (Scheme 7) was used for the encapsulation of the hybrid perovskite quantum dots (POSS-PQD, size~40 nm). They were subsequently applied for aptamers labeling (POSS-PQDs-Apt) that could operate in the aquaculture environment [59].

The encapsulation of PQD with POSS did not change the position (519 nm) and intensity of the PL peak. The hybrid particles were hydrolytically stable and only a 10% decrease in the PL intensity was observed after the immersion of POSS-PQD into water for 24 h. POSS-PQDs-Apt were used as a signal probe to prepare a composite turn-on aptasensor containing titanium carbide (Ti_3_C_2_) MXene particles as fluorescence quenchers. The device was used for screening pathogenic bacteria (*Vibrio parahaemolyticus*) in seawater.

Analogously, a fluorescent aptasensor based on DNA aptamer-functionalized multicolor polyhedral oligomeric silsesquioxane-perovskite quantum dots (cDNA-POSS-PQDs) (constructed with the use of octaaminophenyl-POSS and immobilized on Ti_3_C_2_ MXene) was developed for the on-site detection of live *Salmonella typhimurium* and *Vibrio parahaemolyticus* in water [60].

## 3. Conclusions

Polyhedral oligomeric silsesquioxanes can be effectively used for the modification of inorganic cesium-halide perovskites CsPbX_3_ (X = Cl, Br, I), both nanocrystals and thin films. Depending on the chemical structure of POSS, they can act as an interlayer component of perovskite structures (ionic octafunctional POSS) or as a passivating and structure-controlling agent of perovskite nanocrystals (bifunctional polyhedral silsesquioxanes of (iBuSiO_3/2_)_7_(R′SiO_3/2_)_1_ type). The literature reports show that the crystallization kinetics of perovskites can be modified by the presence of structure-directing POSS. This approach results in a reduction in the number of crystallographic defects in the perovskite-based materials and, consequently, an improvement/modification in their optoelectronic properties. In addition, POSS can enhance the hydrolytic and thermal stability of perovskite structures.

The reason why POSS-perovskites are still somewhat underdeveloped and even slightly abandoned in this branch of materials chemistry is the fact that quite satisfactory results can be obtained by using simple trialkoxysilanes for the hydrophobization of perovskite coatings. In this context, octahedral POSS ligands, which are not as readily available as their R′Si(OR)_3_ counterparts, seem to be a slightly more complicated solution. However, because POSS are widely sought after in materials chemistry, high-throughput, low-cost synthesis protocols for functionalized silsesquioxane cubes are constantly being developed and improved. It should also be emphasized that, usually, only very small amounts of POSS are needed to achieve an outstanding effect of changing the structure and properties of perovskites. Moreover, the use of higher concentrations is not recommended due to the insulating effect of silsesquioxane cages. These reasons allow for us to hope for a renewed interest in the use of POSS for thin-film solar cells, either in the form of nanocrystal components or passivating layers.

Hybrid materials formed from the combination of POSS and perovskites appear to have an even larger potential in materials chemistry. These nanoscale structures are currently being intensively studied for a wide range of possible applications. Efficient light-emitting diodes, luminescent inks, and light absorbers in solar cells with increased lifetime are the immediate answer. However, POSS/perovskite hybrids have also been used as fluorescent probes and aptasensors that operate in situ in aqueous environments. We hope that inciting interest in the hybrid combinations of polyhedral silsesquioxanes and perovskite structures will result in embracing challenges, which will lead to new, interesting solutions in this field, going beyond the already beaten paths.
